# Characteristics of Australian and New Zealand osteopaths who treat patients presenting with non-musculoskeletal complaints: outcomes from two practice-based research networks

**DOI:** 10.1186/s12998-025-00598-9

**Published:** 2025-08-23

**Authors:** Brett Vaughan, Francesco Cerritelli, Jerry Draper-Rodi, Jack Feehan, Ana Paula A. Ferreira, Michael Fleischmann, Gopi McLeod, Cindy McIntyre, Chantal Morin, Lee Muddle, Oliver P. Thomson, Loïc Treffel, Nicholas Tripodi, Kesava Kovanur Sampath, Niklas Sinderholm Sposato, Amie Steel, Jon Adams

**Affiliations:** 1 School of Public Health, University of Technology Sydney, Sydney, Australia; 2 Faculty of Health, Southern Cross University, Lismore, Australia; 3 School of Medical and Health Sciences, Edith Cowan University, Perth, Australia; 4 Department of Medical Education, University of Melbourne, Melbourne, Australia; 5 NYIT College of Osteopathic Medicine, Old Westbury, NY USA; 6 Foundation COME Collaboration, Pescara, Italia; 7 Health Sciences University, London, UK; 8 National Council for Osteopathic Research, London, UK; 9 School of Health and Biomedical Sciences, RMIT, Melbourne, Australia; 10 Instituto Brasileiro de Osteopatia IBO, Rio de Janeiro, Brasil; 11 College of Sport, Health and Engineering, Victoria University, Melbourne, Australia; 12 School of Rehabilitation, Faculty of Medicine and Health Sciences, Université de Sherbrooke, Quebec, Canada; 13 Institut Toulousain d’Ostéopathie Pôle Recherche, ITO-IRF’O, Toulouse, France; 14 Institute of Health and Sport, Victoria University, Melbourne, Australia; 15 Centre for Health and Social Practice, Waikato Institute of Technology, Te Pukenga, Hamilton, New Zealand; 16 Centre of Excellence in Manual and Manipulative Therapy, Duke University, Durham, United States of America; 17 Sahlgrenska Academy, University of Gothenburg, Gothenburg, Sweden

**Keywords:** Allied health, Manual therapy, Manipulative therapy, Osteopathic medicine, Public health

## Abstract

**Background:**

Australian and New Zealand osteopaths predominantly manage musculoskeletal complaints using a variety of modalities including manual therapy, exercise and lifestyle and occupational advice. There appears to be a small percentage of patients who seek osteopathy care for non-musculoskeletal issues such as conditions affecting the gastrointestinal tract. The evidence base for osteopathic treatment as part of the management of such conditions is equivocal. The aim of this study was to describe the practice of Australian and New Zealand osteopaths who report *often* treating patients with non-musculoskeletal complaints.

**Methods:**

This study is a secondary analysis of data from the Australian and New Zealand osteopathy practice-based research networks (PBRNs) collected in Australia from July to December 2016 and in New Zealand from August to December 2018. Respondents to the PBRN baseline surveys were asked to provide information about their demographic, patient and clinical management characteristics. One of these characteristics was the frequency of treating patients presenting with non-musculoskeletal complaints. Descriptive and inferential statistics were used to inform regression modelling of significant predictors of *often* managing non-musculoskeletal complaints.

**Results:**

Of the 1254 osteopath participants from Australia and NZ, 13.5% (*n* = 170) reported *often* treating patients presenting with non-MSK complaints. Significant predictors of *often* treating patients presenting with non-MSK complaints were often using visceral (ORa 3.54 95%CI 2.15–5.85) and Osteopathy in the Cranial Field (OCF) (ORa 2.05 95%CI 1.20–3.51) techniques, and often treating patients up to the age of 3 years (ORa 3.05 95%CI 1.89–4.90).

**Conclusion:**

More than one in ten Australian and New Zealand osteopaths report *often* treating patients presenting with non-MSK complaints, with the dominant manual therapy approaches used being visceral techniques and OCF. This study provides a unique insight into the characteristics of osteopaths who often treat patients presenting with non-MSK complaints. Further research is required to examine if patients seek out care from an osteopath specifically for non-MSK complaints or primarily seek out care from an osteopath for MSK complaints but are managed for non-MSK complaints as a secondary consideration.

## Background

Osteopathy is a primary care profession in Australia and New Zealand (NZ) [1], meaning patients can directly seek care for a variety of complaints without referral from a medical practitioner. While most patients in Australia and NZ present to osteopaths for musculoskeletal (MSK) issues [2–5], patients may also consult an osteopath for non-musculoskeletal (non-MSK) complaints that may be affected by co-existing MSK issues. A systematic review of international surveys profiling the practice of osteopaths found the third most common presenting complaint to an osteopath was related to obstetrics, gynaecological and pregnancy-related problems [2]. In the United Kingdom, where osteopathy practice is similar to Australia and NZ [2], 8.5% of patients’ main complaints are non-MSK in nature [6], with similar rates reported in other countries [7].

With roots in the late 19th century, the philosophy of osteopathy claims a ‘holisitc’ approach to healthcare [8] whereby the structure and function of bodily systems are implicated in the development of pain/illness and subsequent treatment [8]. Osteopathic principles propose the body has innate healing mechanisms, that when optimised by manual therapy techniques promote self-healing, and contribute to overall well-being [9]. Descriptions of practice suggest some (but not all) osteopaths use this principle, along with the concept of an interrelationship between the body’s structure and function, to help guide their assessment and treatment [10, 11]. However, there is emerging commentary as to the weight osteopaths should place on these principles and concepts [12] given the need for additional research on care outcomes [13].

High-level evidence for the efficacy of osteopathic management for non-MSK conditions is limited [14, 15] and evidence for techniques that might be used by osteopaths for the management of non-MSK conditions have been reported to have little effect [16, 17]. However, it is unlikely that osteopaths use only one technique/approach and address other areas of the body when managing non-MSK complaints [18]. There is emerging evidence that suggests osteopathic care can assist in the management of several non-MSK complaints including irritable bowel syndrome [19], gastro-oesophageal reflux [20], and menstrual pain [21] using approaches directed towards both musculoskeletal and non-musculoskeletal structures, and combined with education/advice [22]. The methodological quality of these studies is variable supporting the need for further research to understand how practitioners manage non-MSK complaints and the potential outcomes of this care. The limited evidence for the treatment of non-MSK complaints by osteopaths continues to fuel ongoing debate, both from within and outside the profession, about evidence-based practices, the scope of osteopathic care, and patient outcomes and satisfaction [12, 23–25]. Given the literature identifies that osteopaths engage in the treatment of patients presenting with non-MSK complaints, the aim of this study is to present data describing the characteristics of osteopaths in Australia and NZ who report *often* providing non-MSK patient care as part of their practice.

## Methods

This study is a secondary analysis of data gathered through two national practice-based research networks (PBRNs) dedicated to the Australian and NZ osteopathy professions [3, 26]. Ethical approval for this study was granted by the University of Technology Sydney, Sydney Human Research Ethics Committee (Australian PBRN: #2014000759; NZ PBRN: #ETH19-3435), and all participating osteopaths provided informed consent.

### Participants

The participants for this study were drawn from the Australian and NZ osteopathy PBRNs. The Osteopathy Research and Innovation Network (ORION), a PBRN for the Australian osteopathy profession, was established and data were collected from July to December 2016. Previous research by Adams, Sibbritt [3] has detailed the nationally representative nature of this data for the profession in terms of gender, age, and practice location. In total, 992 osteopaths completed the ORION questionnaire, resulting in a response rate of 49.1%. The PBRN for NZ osteopaths is the Osteopathy Research Connect-NZ (ORC-NZ) PBRN which was established in 2018, with participants recruited from August to December 2018. In total, 277 osteopaths completed the ORC-NZ survey with 253 osteopaths agreeing to join the PBRN, resulting in a response rate of 48.7% [26]. Data from the full sample of respondents for both Australia and NZ were pooled and analysed for this secondary analysis.

### Questionnaire

The ORION and ORC-NZ questionnaires were developed specifically for the establishment of the respective PBRNs and were designed using expert consensus of practicing osteopaths [3, 26]. The questionnaires were designed to enable collection of a range of data relevant to developing a better understanding of the workforce and the practice of osteopathy. The questionnaires were piloted in their respective jurisdictions. Questionnaire items included demographics such as age, gender, qualifications, and years of experience in practice. The questionnaire also covered practice details including the average number of hours dedicated to patient care, the number of patients seen weekly, whether other healthcare professionals worked at their workplace, and referrals to or from other healthcare professionals. Regarding patient management, participants indicated the frequency of treating different body regions, the use of specific techniques and adjunctive therapies, and the frequency of managing different patient groups.

### Outcome variable and exposure variables

The primary outcome variable in this study was frequency of managing “non-musculoskeletal complaints”. Participants rated their frequency of managing non-MSK complaints on a four-point scale: never, rarely, sometimes, and often. The first three options were collapsed to “not often” whilst “often” was treated as the other binary outcome. The exposure variables in this study encompassed information related to the previously mentioned demographic and practice characteristics, which were collected using either frequency, binary (yes/no) responses or continuous data. Missing data for binary variables was treated as missing at random and imputed as “not often” using simple imputation [27]. Less than 0.5% of data for an individual binary item was missing and simple imputation was deemed appropriate [28]. It was determined that the respondents who did not respond to a frequency item were unlikely to respond “often” as it was likely the item was not meaningful or relevant to their practice.

### Open text responses

Respondents were given the option to provide the type of non-MSK complaints they manage in clinical practice through an open-text response. Responses were extracted and sorted by body system by two authors (MF and KSK) who cross-checked their analysis to ensure consistency, for example, respiratory or gastrointestinal. Where open text responses were ambiguous or did not fit a category, these responses were treated as ‘other’.

### Statistical analysis

Inferential statistics were employed to explore the relationships between individual practice characteristics and the frequency of managing non-MSK complaints. Frequency responses were subjected to independent measure t-tests, while binary responses underwent chi-square tests with continuity correction. Variables with a significance level of *p* < 0.10 were selected and subsequently entered into a multivariate logistic regression model. Backward elimination was utilised to identify the key predictors of osteopaths who report *often* manage non-MSK complaints. Adjusted odds ratios (ORa) with 95% confidence intervals (CI) and p-values were computed from this regression modelling. Statistical significance was set at *p* < 0.05, and inferential statistical analyses were conducted using JASP (version 0.18) [29], while regression modelling was carried out using SPSS (version 27).

## Results

Data from 1254 survey participants were analysed, of whom 170 (13.5%) Australian and NZ osteopaths reported they *often* treat non-MSK complaints, whilst 1087 osteopaths reported treating non-MSK complaints *not often*. With respect to demographic characteristics, practitioners who reported *often* treating non-MSK complaints were older (with an average of 44.7 years old), had more experience (with an average of 16 years in clinical practice) and had various levels of qualification with fewer holding a diploma, advanced diploma, bachelor’s degree, PhD, or other qualifications and more holding a master’s degree. They were also more involved in teaching and volunteering work than osteopaths who did *not often* treat these complaints (Table 1). The gender ratio was similar between both groups.

**Table 1:** Demographic characteristics of Australian and New Zealand osteopaths who report *often* versus *not often* treating non-musculoskeletal complaints

	Often		Not often			*p*-value
**Gender (n, %)**						
Male (*n* = 701)	92 (54.2%)		609 (56.0%)			0.82
Female (*n* = 555)	78 (45.8%)		477 (43.8%)			
Other (*n* = 1)	0		1 (0.1%)			
**Age (years**,** n = 1254)**						
Mean (± SD)	44.7 (± 11.3)		38.7 (± 11.3)			< 0.01^*^
**Years in clinical practice (n = 1239)**						
Mean (± SD)	16.2 (± 10.0)		11.5 (± 9.2)			< 0.01^^^
**Patient care hours per week (n = 1251)**						
Mean (± SD)	29.2 (± 11.6)		27.7 (± 11.9)			0.12
**Patient visits per week (n = 1050)**						
Mean (± SD)	37.6 (± 21.3)		35.1 (± 19.3)			0.14
**Country of practice (n = 1257)**						
Australia	126 (74.2%)		854 (78.6%)			0.23
New Zealand	44 (25.9%)		233 (21.4%)			
**Qualification (n**,** %)**						
Diploma	82 (7.6%)		21 (12.3%)			< 0.01
Advanced diploma	10 (0.9%)		2 (1.2%)			
Bachelor’s degree	246 (22.7%)		42 (24.7%)			
Master’s degree	717 (66.3%)		85 (50.0%)			
PhD	3 (0.3%)		3 (1.7%)			
Other	24 (2.2%)		17 (10.0%)			
**Involved in as an osteopath (n**,** %)**						
University teaching	28 (16.5%)		112 (10.3%)			0.02^#^
Clinical supervision (students)	31 (18.2%)		141 (13.0%)			0.06
Research	15 (8.8%)		60 (5.5%)			0.13
Volunteer	45 (26.5%)		162 (14.9%)			< 0.01^@^

* d = 0.53 95%CI[0.36–0.69]; ^ d = 0.50 95%[0.34–0.67]; # OR = 1.72 95%CI[1.09–2.70]; @ OR = 2.05 95%CI[1.41-3.00]

Osteopaths in NZ and Australia who report *often* treating patients with non-MSK complaints were more likely to be co-located with a naturopath (OR 2.18) and a psychologist (OR 1.67), than osteopaths who *not often* treat these complaints. With respect to sending referrals to other health professionals, Australian and NZ osteopaths who report *often* treating patients with non-MSK complaints were more likely to send referrals to a psychologist (OR 2.67), a naturopath (OR 1.83), a nutritionist (OR 1.59) and an acupuncturist (OR 1.48), and less likely to send referrals to podiatrist (OR 0.60) compared with osteopaths who do not *often* treat patients with non-MSK complaints (*p* < 0.05) (Table 2). Regarding receiving referrals from other health professionals, Australian and NZ osteopaths who report *often* treating patients with non-MSK complaints were more likely to receive referrals from a psychologist (OR 3.81), an occupational therapist (OR 2.72), a naturopath (OR 2.31), another osteopath (OR 2.24), a physiotherapist (OR 1.97), or an acupuncturist (OR 1.51), compared with osteopaths who do not *often* treat non-MSK complaint patients (*p* < 0.05) (Table 2). With respect to patient assessment, Australian and NZ osteopaths who report *often* treating patients with non-MSK complaints were more likely to use cranial nerve testing (OR 1.99) and less likely to use orthopaedic testing (OR 0.39) compared with osteopaths who do not *often* treat patients with non-MSK complaints (*p* < 0.05) (Table 2).

**Table 2:** Practice characteristics of Australian and new Zealand osteopaths who report *often* versus *not often* treating non-musculoskeletal complaints

	Often	Not often		*p*-value		OR [95% CI]	
**Practice location**							
Urban practice	145 (85.3%)	908 (83.5%)		0.64		1.14 [0.72–1.80]	
More than one practice location	64 (37.6%)	373 (34.3%)		0.44		1.15 [0.83–1.61]	
**Co-located with other health professionals (‘yes’)**							
Osteopath	104 (61.1%)	683 (62.8%)		0.74		0.93 [0.67–1.30]	
General practitioner	15 (8.8%)	76 (7.0%)		0.48		1.27 [0.72–2.30]	
Specialist medical practitioner	9 (5.3%)	26 (2.4%)		0.06		2.28 [1.05–4.95]	
Podiatrist	21 (12.3%)	141 (13.0%)		0.92		0.94 [0.58–1.54]	
Physiotherapist	29 (17.1%)	153 (14.0%)		0.36		1.25 [0.81–1.94]	
Exercise physiologist	21 (12.3%)	104 (9.6%)		0.32		1.33 [0.81–2.19]	
Occupational therapist	4 (2.3%)	19 (1.7%)		0.81		1.35 [0.45–4.03]	
Psychologist	43 (25.3%)	183 (16.8%)		**0.01**		**1.67 [1.14–2.45]**	
Massage therapist	73 (42.9%)	514 (47.2%)		0.33		0.84 [0.60–1.16]	
Acupuncturist	37 (21.8%)	221 (20.3%)		0.74		1.10 [0.73–1.61]	
Naturopath	50 (29.4%)	174 (16.0%)		**< 0.01**		**2.18 [1.51–3.16]**	
Dietician	13 (7.7%)	65 (6.0%)		0.50		1.30 [0.70–2.41]	
Nutritionist	17 (10.0%)	79 (7.3%)		0.27		1.42 [0.82–2.46]	
**Send referrals to other health professionals (‘yes’)**							
Osteopath	101 (59.4%)	570 (52.5%)		0.11		1.33 [0.95–1.84]	
General practitioner	152 (89.4%)	961 (88.4%)		0.80		1.11 [0.65–1.87]	
Specialist medical practitioner	82 (48.2%)	564 (51.9%)		0.42		0.86 [0.62–1.19]	
Podiatrist	83 (48.8%)	667 (61.3%)		**< 0.01**		**0.60 [0.43–0.83]**	
Physiotherapist	60 (35.3%)	393 (36.1%)		0.89		0.96 [0.68–1.35]	
Exercise physiologist	60 (35.3%)	354 (32.6%)		0.53		1.13 [0.80–1.58]	
Occupational therapist	20 (11.8%)	110 (10.1%)		0.60		1.18 [0.71–1.96]	
Psychologist	93 (54.7%)	338 (31.1%)		**< 0.01**		**2.67 [1.91–3.72]**	
Massage yherapist	115 (67.7%)	726 (66.8%)		0.89		1.04 [0.73–1.47]	
Acupuncturist	100 (58.8%)	535 (49.2%)		**0.02**		**1.48 [1.06–2.05]**	
Naturopath	101 (59.4%)	482 (44.3%)		**< 0.01**		**1.83 [1.32–2.55]**	
Dietician	32 (18.8%)	155 (14.3%)		0.15		1.39 [0.91–2.12]	
Nutritionist	34 (20.0%)	148 (13.6%)		**0.04**		**1.59 [1.05–2.40]**	
**Receive referrals from another health professionals (‘yes’)**							
Osteopath	134 (78.8%)	678 (62.4%)		**< 0.01**		**2.24 [1.52–3.30]**	
General practitioner	151 (88.8%)	968 (89.1%)		1.00		0.97 [0.58–1.63]	
Specialist medical practitioner	49 (28.8%)	275 (25.3%)		0.38		1.19 [0.83–1.71]	
Podiatrist	71 (41.7%)	449 (41.3%)		0.97		1.02 [0.73–1.41]	
Physiotherapist	75 (44.1%)	311 (28.6%)		**< 0.01**		**1.97 [1.41–2.74]**	
Exercise physiologist	44 (25.8%)	227 (20.9%)		0.17		1.32 [0.91–1.92]	
Occupational therapist	24 (14.2%)	62 (5.7%)		**< 0.01**		**2.72 [1.64–4.49]**	
Psychologist	60 (35.3%)	136 (12.5%)		**< 0.01**		**3.81 [2.65–5.48]**	
Massage therapist	136 (80.0%)	808 (74.3%)		0.13		1.38 [0.92–2.06]	
Acupuncturist	83 (48.8%)	420 (38.7%)		**0.01**		**1.51 [1.10–2.10]**	
Naturopath	97 (57.0%)	397 (36.5%)		**< 0.01**		**2.31 [1.66–3.21]**	
Dietician	35 (3.2%)	11 (6.5%)		0.06		2.08 [1.03–4.18]	
Nutritionist	15 (8.8%)	59 (5.4%)		0.11		1.68 [0.93–3.04]	
**Diagnostic imaging**							
Referral for imaging (‘often’)	25 (14.7%)	113 (10.4%)		0.12		1.48 [0.93–2.37]	
Investigation of unknown pathologies	126 (74.2%)	817 (75.2%)		0.84		0.94 [0.65–1.37]	
Investigation of suspected diagnosis	143 (84.2%)	927 (85.3%)		0.78		0.91 [0.59–1.42]	
Investigation of potential fractures	128 (75.3%)	844 (77.6%)		0.56		0.88 [0.60–1.30]	
Rule out risk factors prior to treatment	58 (34.2%)	320 (29.4%)		0.25		1.24 [0.88–1.75]	
General screening of the spine	10 (5.9%)	44 (4.1%)		0.37		1.48 [0.73-3.00]	
**Patient assessment (‘yes’)**							
Orthopaedic testing	161 (94.7%)	1064 (97.8%)		**0.03**		**0.39 [0.17–0.85]**	
Clinical assessment algorithm	64 (37.6%)	484 (44.5%)		0.11		0.75 [0.54–1.05]	
Neurological testing	157 (92.3%)	1013 (93.1%)		0.81		0.88 [0.48–1.63]	
Screening questionnaire	112 (65.8%)	758 (69.7%)		0.35		0.84 [0.59–1.18]	
Cranial nerve testing	135 (79.4%)	717 (65.9%)		**< 0.01**		**1.99 [1.34–2.95]**	

Values in bold are statistically signifcant

Osteopaths who report *often* treating patients with non-MSK complaints were more likely to report often discussing diet/nutrition (OR 3.05), nutritional supplements (OR 2.81), stress (OR 2.31), and smoking (OR 2.23) with patients, compared with osteopaths who do *not often* treat patients with non-MSK complaints (Table 3). Australian and NZ osteopaths who report *often* treating patients with non-MSK complaints were more likely to see patients from these groups: post-surgery (OR 6.61), below three years of age (OR 6.98), between four and 18 years of age (OR 4.54), older adults (OR 1.51), pregnant people (OR 3.02), and non-English speaking people (OR 2.66), compared with osteopaths who do *not often* treat patients with non-MSK complaints (Table 3). Australian and NZ osteopaths who report *often* treating patients with non-MSK complaints were more likely to report often treating a wide range of conditions compared with osteopaths who do *not often* treat non-MSK complaints (Table 3).

**Table 3:** Clinical management characteristics of Australian and new Zealand osteopaths who report *often* versus *not often* treating non-musculoskeletal complaints

	Often	Not often	*p*-value	OR [95%CI]	
**Discuss with patients (‘often’)**					
Diet/nutrition	105 (61.7%)	376 (34.6%)	**< 0.01**	**3.05 [2.18–4.26]**	
Smoking/alcohol/drugs	48 (28.2%)	163 (15.0%)	**< 0.01**	**2.23 [1.54–3.24]**	
Physical activity	145 (85.3%)	966 (88.9%)	0.22	0.72 [0.46–1.15]	
Occupation health & safety	79 (46.4%)	535 (49.3%)	0.54	0.89 [0.64–1.23]	
Pain counselling	37 (21.7%)	282 (25.9%)	0.28	0.79 [0.52–1.18]	
Stress	115 (67.7%)	516 (4.6%)	**< 0.01**	**2.31 [1.63–3.25]**	
Nutritional supplements	76 (44.7%)	243 (22.3%)	**< 0.01**	**2.81 [2.01–3.92]**	
Medication	68 (40.0%)	419 (38.5%)	0.79	1.06 [0.76–1.48]	
**Patient subgroups (treat ‘often’)**					
Up to 3 years of age	87 (51.5%)	143 (13.2%)	**< 0.01**	**6.98 [4.92–9.90]**	
4 to 18 years of age	100 (58.8%)	260 (23.9%)	**< 0.01**	**4.54 [3.24–6.35]**	
Older adults	116 (68.2%)	637 (58.6%)	**0.02**	**1.51 [1.07–2.14]**	
Pregnancy	96 (56.5%)	326 (30.0%)	**< 0.01**	**3.02 [2.17–4.21]**	
Non-English speaking	13 (7.7%)	33 (3.0%)	**< 0.01**	**2.66 [1.37–5.16]**	
Sport-related injuries	87 (51.5%)	548 (50.5%)	0.87	1.04 [0.75–1.44]	
Post-surgery	51 (30.0%)	66 (6.1%)	**< 0.01**	**6.61 [4.38–9.98]**	
Indigenous populations	10 (5.8%)	38 (3.5%)	0.20	1.72 [0.84–3.52]	
**Conditions treated (‘often’)**					
Neck pain	164 (96.4%)	1068 (98.2%)	0.21	0.48 [0.19–1.23]	
Thoracic pain	153 (90.0%)	992 (91.3%)	0.70	0.86 [0.50–1.48]	
Back pain	166 (97.6%)	1070 (98.5%)	0.60	0.62 [0.20–1.88]	
Hip pain	138 (81.7%)	782 (71.9%)	**0.01**	**1.74 [1.15–2.62)**	
Knee pain	125 (73.5%)	495 (45.6%)	**< 0.01**	**3.31 [2.31–4.75]**	
Ankle pain	109 (64.2%)	323 (29.7%)	**< 0.01**	**4.22 [3.00-5.93]**	
Foot pain	102 (60.0%)	266 (24.5%)	**< 0.01**	**4.63 [3.31–6.48]**	
Shoulder pain	147 (86.5%)	868 (79.92%)	0.05	1.60 [1.01–2.55]	
Elbow pain	81 (47.9%)	231 (21.3%)	**< 0.01**	**3.40 [2.43–4.75]**	
Wrist pain	74 (43.5%)	162 (14.9%)	**< 0.01**	**4.40 [3.11–6.21]**	
Hand pain	54 (31.9%)	100 (9.2%)	**< 0.01**	**4.61 [3.14–6.77]**	
Postural disorders	120 (70.6%)	692 (63.7%)	0.10	1.37 [0.96–1.94]	
Degenerative spine disorders	111 (65.3%)	610 (56.2%)	**0.03**	**1.46 [1.05–2.06]**	
Headache	159 (93.5%)	960 (88.3%)	0.06	1.91 [1.01–3.62]	
Migraine disorders	110 (64.7%)	394 (36.3%)	**< 0.01**	**3.21 [2.29–4.51]**	
Spine health maintenance or prevention	111 (65.3%)	610 (56.2%)	0.03	1.47 [1.05–2.06]	
Chronic or persistent pain	135 (79.4%)	628 (57.8%)	**< 0.01**	**2.81 [1.90–4.16]**	
Tendinopathies	83 (49.1%)	404 (37.2%)	**< 0.01**	**1.63 [1.18–2.26]**	
Temporomandibular joint	76 (45.0%)	142 (13.2%)	**< 0.01**	**5.43 [3.82–7.71]**	
**Manual therapy (use ‘often’)**					
Counterstrain	70 (41.2%)	415 (38.3%)	0.52	1.13 [0.81–1.57]	
Muscle energy technique	98 (57.7%)	846 (77.9%)	**< 0.01**	**0.39 [0.27–0.54]**	
High-velocity, low-amplitude manipulation	71 (41.8%)	720 (66.3%)	**< 0.01**	**0.36 [0.26–0.51]**	
Peripheral joint manipulation	75 (44.2%)	462 (42.6%)	0.77	1.06 [0.76–1.47]	
Soft tissue technique	116 (68.2%)	961 (88.5%)	**< 0.01**	**0.28 [0.19–0.40]**	
Myofascial release	112 (65.8%)	657 (60.5%)	0.21	1.26 [0.89–1.76]	
Visceral techniques	71 (41.7%)	92 (8.5%)	**< 0.01**	**7.75 [5.34–11.24]**	
Lymphatic pump	43 (25.3%)	67 (6.2%)	**< 0.01**	**5.15 [3.37–7.88]**	
Autonomic balancing	84 (49.7%)	127 (11.7%)	**< 0.01**	**7.46 [5.23–10.63]**	
Biodynamics	70 (41.2%)	13 (12.2%)	**< 0.01**	**5.02 [3.52–7.15]**	
Functional technique	91 (53.5%)	298 (27.4%)	**< 0.01**	**3.04 [2.19–4.23]**	
Balanced ligamentous tension	110 (64.7%)	366 (33.7%)	**< 0.01**	**3.61 [2.57–5.06]**	
Chapman’s reflexes	9 (5.3%)	26 (2.4%)	0.06	2.28 [1.05–4.95]	
Trigger point therapy	40 (23.6%)	295 (27.2%)	0.36	0.82 [0.56–1.20]	
Osteopathy in the cranial field	115 (67.5%)	253 (23.3%)	**< 0.01**	**6.88 [4.84–9.77]**	
Facilitated positional release	59 (34.9%)	174 (16.1%)	**< 0.01**	**2.81 [1.97-4.00]**	
Dry needling	19 (11.2%)	226 (20.8%)	**< 0.01**	**0.48 [0.29–0.79]**	
Exercise prescription	120 (70.6%)	824 (75.9%)	0.16	0.76 [0.53–1.09]	
Shockwave therapy	3 (1.7%)	17 (1.6%)	1.00	1.13 [0.33–3.89]	
Ultrasound	1 (0.6%)	26 (2.4%)	0.22	0.24 [0.03–1.78]	
TENS	3 (1.7%)	23 (2.1%)	0.99	0.83 [0.25–2.79]	
Instrument-assisted soft-tissue	3 (1.7%)	11 (1.0%)	0.63	1.75 [0.48–6.35]	
Sport taping	18 (10.6%)	131 (12.1%)	0.67	0.86 [0.51–1.45]	

Values in bold are statistically signifcant

Australian and NZ osteopaths who report *often* treating patients with non-MSK complaints were more likely to use manual therapy strategies such as visceral techniques (OR 7.75), autonomic balancing (OR 7.46), osteopathy in the cranial field (OCF) (OR 6.88), lymphatic pump techniques (OR 5.15), biodynamics (OR 5.02), balanced ligamentous tension techniques (OR 3.61), functional techniques (OR 3.04) or facilitated positional release (OR 2.81) compared with osteopaths who do *not often* treat patients with non-MSK complaints (Table 3). In parallel, Australian and NZ osteopaths who report *often* treating patients with non-MSK complaints were less likely to use manual therapy strategies such as dry needling (OR 0.48), muscle energy technique (OR 0.39), high-velocity low-amplitude manipulation (OR 0.36), and soft tissue technique (OR 0.28) compared with osteopaths who do *not often* treat patients with non-MSK complaints (Table 3).

Significant predictors of Australian and NZ osteopaths *often* treating patients with non-MSK complaints included *often* treating patients under the age of three years (ORa 3.05) and *often* treating patients with chronic or persistent pain (ORa 2.12) (Table 4). With respect to manual therapy strategies, significant predictors of Australian and NZ osteopaths *often* treating patients with non-MSK complaints were *often* using OCF (ORa 2.05) and manual therapy techniques applied to the viscera (ORa 3.54) (Table 4).

**Table 4:** Statistically significant variables and their associated adjusted odds ratios for Australian and new Zealand osteopaths who report *often* treating non-musculoskeletal complaints

		95% Confidence interval		
	Adjusted OR	Lower	Upper	*p*-value
Age	1.03	1.00	1.05	0.007
Send referrals to a podiatrist (yes)	0.57	0.37	0.87	0.010
Send referrals to an acupuncturist (yes)	0.62	0.39	0.97	0.039
Receive referrals from a physiotherapist (yes)	1.66	1.07	2.58	0.024
Treat patients up to age 3 years (often)	3.05	1.89	4.90	< 0.001
Treat patients with hip musculoskeletal complaints (often)	0.33	0.17	0.63	0.001
Treat patients with knee musculoskeletal complaints (often)	1.91	1.02	3.57	0.042
Treat patients with foot musculoskeletal complaints (often)	1.81	1.07	3.05	0.027
Treat patients with temporomandibular joint disorders (often)	2.01	1.23	3.28	0.005
Treat patients with chronic or persistent pain (often)	2.12	1.27	3.56	0.004
Treat patients with migraine (often)	1.75	1.10	2.79	0.018
Discuss diet (often)	1.99	1.27	3.14	0.003
Discuss nutritional supplements (often)	1.76	1.13	2.75	0.013
Use autonomic balancing (often)	2.11	1.29	3.46	0.003
Use Osteopathy in the Cranial Field (OCF) techniques (often)	2.05	1.20	3.51	0.009
Use visceral techniques (often)	3.54	2.15	5.83	< 0.001

OR: Odds ratio

**Fig. 1:**
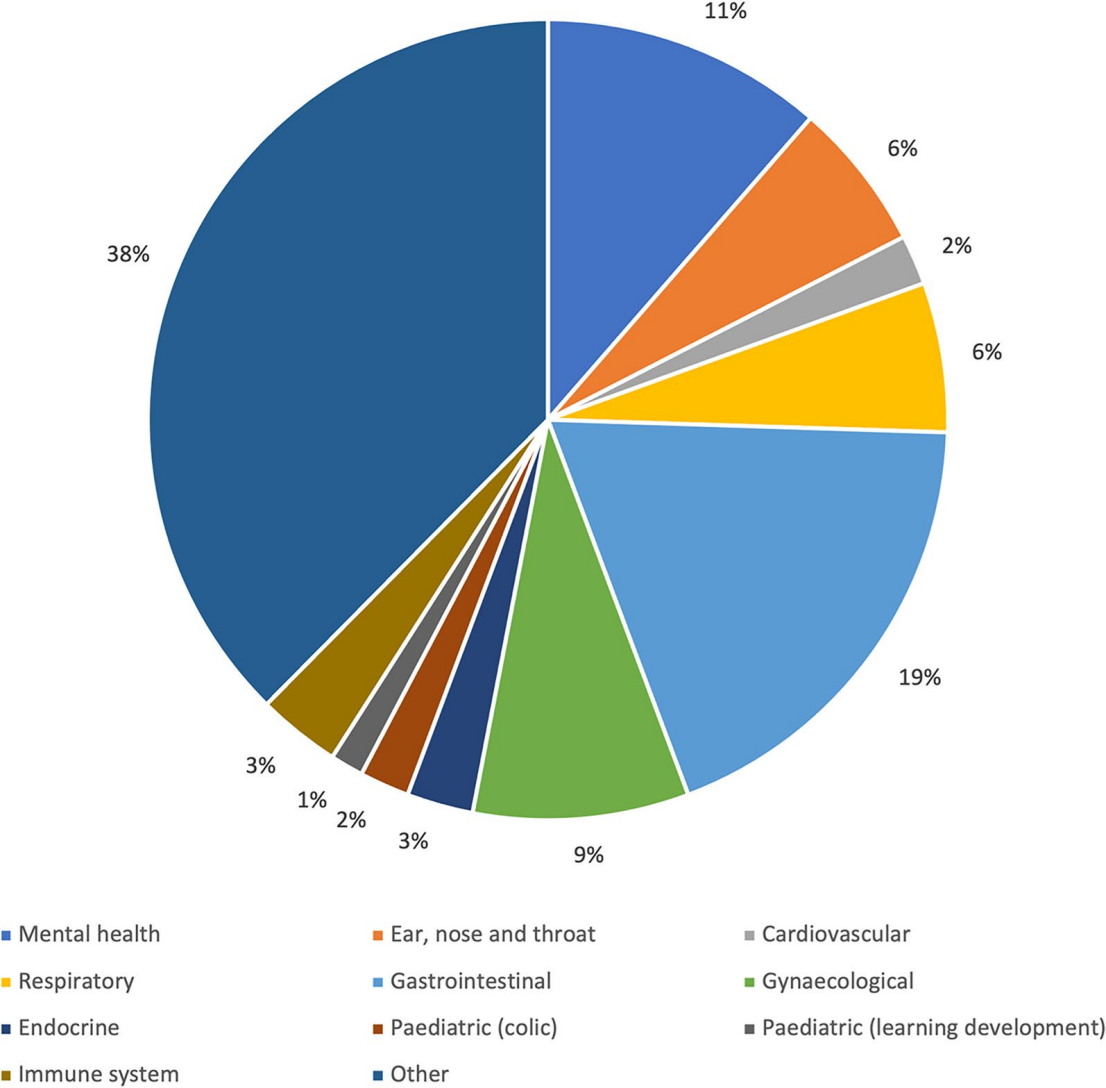
Classification of conditions reported being treated by Australian and New Zealand osteopaths who report *often* treating patients with non-musculoskeletal complaints

A graphical summary of the open-text responses where respondents could indicate the conditions they manage in clinical practice is presented in Fig. 1. Sixty-five of the 170 (38.2%) respondents provided open-text responses. Australian and NZ osteopaths who report *often* treating patients with non-MSK complaints indicated that they most often treated patients’ mental health and gastrointestinal complaints (Fig. 1). For ‘other’ (Fig. 1), respondents typically indicated “visceral” or variations of the same.

## Discussion

While MSK complaints are the most common reason patients seek osteopathic treatment in Australian and NZ [4, 5], 13.5% of osteopaths in these two countries report *often* treating non-MSK complaints in practice. Results observed in the current work are consistent with other countries where around 10% of patient presentations to osteopaths would be classified as non-MSK [2]. Our secondary analysis of data from two national osteopathy PBRNs provide further insight into the management of non-MSK complaints by Australian and NZ osteopaths, and their associated demographic and practice characteristics.

Our analysis shows reporting *often* using visceral techniques and OCF were significant predictors of Australian and NZ osteopaths *often* treating patients presenting with non-MSK complaints. Visceral techniques in osteopathic practice are the application of manual pressure to the tissues associated with the viscera with the goal of improving physiologic function [30]. Osteopaths are reported to include visceral techniques in the treatment of MSK complaints such as neck [31] and low back pain [30, 32], and in non-MSK complaints such as irritable bowel syndrome [33], menstrual pain [21] and gastro-oesophageal reflux [20, 34]. There are, however, conflicting views as to the place of visceral techniques in osteopathic practice [18] and limited evidence of its effectiveness when used alone [14, 35, 36]. This gap between practice and current evidence may explain why osteopaths who often use visceral techniques, compared with those who use these techniques less frequently, report greater value of research informing patient care [37]; highlighting the need for further evidence to guide practice.

Similar to visceral osteopathy, OCF has been claimed to help complaints commonly presenting to osteopaths such as migraines [38], temporomandibular joint (TMJ) pain [39], and for non-MSK complaints such as vestibular pathology [40], cerebral palsy [41], traumatic brain injury (Dickerson et al., 2022), and infantile colic [42]. Consistent with visceral techniques, the evidence base for the effects of OCF used in isolation, is limited [43]. Although both visceral techniques and OCF are used in the treatment of non-MSK complaints by some osteopaths, there is a need to better understand whether they can be a useful adjunct to usual care [44].

Treating TMJ complaints and migraines *often* were identified as significant predictors of Australian and NZ osteopaths *often* treating patients with non-MSK complaints in our study. Osteopathic care for TMJ complaints has been shown to positively impact pain and function and health-related quality of life [39], and reduce medication use [45]. The link between non-MSK complaints and treatment of TMJ complaints is less clear but may be related to the whole-body treatment philosophy espoused by osteopaths. It may also be that the TMJ is seen as a key area for treatment related to non-MSK complaints given its reported influence on posture [46], for example, or potentially related to the use of OCF described above. Migraines and their association with *often* treating non-MSK complaints may be related to the reported ‘gut-brain’ link [47, 48] and potential presence of visceral symptoms before, during and after migraine episodes [49]. Osteopaths may be directing treatment to these non-MSK symptoms or treating visceral structures to assist in the management of migraines. The methods through which osteopaths are incorporating the management of non-MSK complaints with care of patients with migraines or TMJ complaints requires additional exploration to understand the association observed, particularly related to clinical reasoning and patient care outcomes.

Our research shows osteopaths in Australia and NZ who reported *often* treating non-MSK complaints were more likely to report *often* treating patients under three years of age and those between 4 and 18 years of age, compared with osteopaths who do *not often* treat non-MSK complaints. This finding aligns with other international osteopathic practice profiles suggesting care for these two age groups includes MSK and non-MSK complaints [2, 50]. From the perspective of non-MSK complaints, there is evidence supporting positive effects of osteopathic treatment for gross motor function in cerebral palsy and sleep improvement and reduction in crying associated with infantile colic [51]. There is also varying levels of evidence supporting osteopathic care of asthma, attention deficit hyperactivity disorder (ADHD), infant feeding issues, and speech disorders in younger populations [51]. Given the breadth of conditions reported in the literature, it is possible Australian and NZ osteopaths who report *often* treating non-MSK complaints in these age groups are providing care for these conditions/issues. As such, it is important to develop a clearer picture of the conditions/issues treated, the outcomes from this care outside of the research setting, and whether osteopaths are participating in multidisciplinary care for various non-MSK conditions/issues.

Often treating patients with chronic pain was a significant predictor of Australian and NZ osteopaths *often* treating non-MSK complaints. This finding may reflect one or all three of the following: the non-MSK complaints osteopaths treat are chronic in nature; chronic pain is being managed by osteopaths as part of a non-MSK complaint; or osteopaths are managing the non-MSK manifestations of chronic pain. The ICD-11 potentially provides insight to these permutations through the chronic secondary non-MSK [52] and chronic secondary visceral pain [53] diagnoses. According to Aziz, Giamberardino [53] chronic primary visceral pain likely co-occurs with other pain conditions, with fibromyalgia being an example of a condition linked to visceral-related issues. Whilst these ICD-11 diagnoses provide an appreciation of the relationship between chronic pain syndromes and visceral-related issues, it is unclear whether it explains the associations found in this current work. The use of the ICD-11 has been advocated in osteopathy as one way to develop a more complete picture of diagnoses and simultaneously improving patient data collection [54]. Further research, including qualitative studies and practice audits, is required to understand the link between *often* treating patients with chronic pain and *often* treating non-MSK complaints where ICD-11 coding could be of value to monitor patient presenting complaints in a standardised manner.

Australian and NZ osteopaths in our study who report *often* treating patients with non-MSK complaints were likely to report *often* discussing diet and nutritional supplements with patients as part of their management. These associations suggest Australian and NZ osteopaths see the need to incorporate these discussions with patients when managing non-MSK disorders [55]. Diet-related issues are reportedly associated with some of the non-MSK conditions osteopaths noted in their open-text comments in the current work. Gastrointestinal complaints were a common issue reported by osteopaths in this secondary analysis, and it is possible incorporating dietary advice for these complaints is a key element of the consultation, particularly as dietary change is advocated for conditions such as IBS [56] and mood disorders [57]. Further research exploring patient adherence and the extent to which dietary advice is provided by Australian and NZ osteopaths for non-MSK complaints would be useful. Similarly, discussions about nutritional supplements are undertaken by Australian and NZ osteopaths who *often* treat non-MSK complaints, which was identified in the current work. Nutritional supplements are advocated for several of the condition’s osteopaths identified in their open-text responses, including IBS [58] and fibromyalgia [59]. It would be helpful to know the extent of these recommendations to accurately inform patient care.

With respect to implications for practice, the current work supports the need to develop further practice-based evidence for the management of non-MSK complaints by osteopaths. Specifically, this evidence should include the collection of patient-reported outcome measure data to explore efficacy and effectiveness. Practitioners should also be cognisant of contemporary evidence for practice, particularly evidence for approaches such as OCF and the application of visceral techniques given their limited evidence base. Engaging with other health professionals, including multi- and inter-professional care, should be considered as routine when managing patients with non-MSK complaints to ensure the patient receives the best available care. Further, practitioners should also be conversant with the latest literature and interventions for non-MSK complaints that are managed in osteopathic practice and engage in continuing professional development to provide safe and effective care.

The current study is constrained by the cross-sectional nature of the questionnaire employed. Questionnaires utilised in such study designs may be vulnerable to biases, including social desirability and acquiescence. The latter bias may impact responses to the non-MSK disorder item on the questionnaire. The real frequency of managing patients with non-MSK complaints may be different to that reported here. The four point Likert-type frequency scale descriptors were not defined in the questionnaire, for example, using a percentage of a day/week. As such, respondents may have attached differing meanings to these descriptors (i.e. often) and this may impact the results. The notion of ‘treatment’ as asked within the questionnaire, may hold varied meanings to osteopaths in the context of their clinical practice. For example, it is plausible, for some osteopaths, treatment is targeted to ameliorating the non-MSK condition (for example Irritable Bowel Syndrome [33]). Others may view their ‘treatment’ as supportive care for a patient experiencing a non-MSK condition (e.g. psychological support or lifestyle advice), or to help patients to manage MSK symptoms (e.g. pain or stiffness) which might be related or secondary to a non-MSK condition and where osteopathy is seen as an adjunct treatment to more conventional care such as in the case of supportive care in cancer [60]. It is also possible that the respondents had differing conceptions of some of the technique approaches (i.e. soft tissue technique, functional technique). These differing conceptions may impact on reported frequency of use of a specific technique approach by an individual respondent. A strength of the current work is the representativeness of the respondents with respect to the demographics of the profession across Australia and NZ. Future research could explore how and why patients may seek out osteopaths to assist in the management of non-MSK complaints, and the clinical reasoning of osteopaths managing non-MSK complaints.

## Conclusion

This study emphasises that a small percentage of osteopaths in Australia and NZ commonly treat patients presenting with non-MSK complaints. Notably, among those who frequently treat patients presenting with non-MSK complaints, there is a prominent use of visceral and OCF techniques. Furthermore, our data indicates non-MSK complaints are being addressed by osteopaths in patients under three years of age and those between 4 and 18 years. The findings raise important questions about whether patients (through their parent/guardian) are seeking care primarily for a non-MSK complaint or care for a MSK complaint with co-existing non-MSK issues. Additionally, factors influencing technique choice, the outcomes associated with care for non-MSK complaints, and guidance regarding supplementation, require exploration. More research is needed to elucidate the decision-making processes underpinning technique selection made by osteopaths for non-MSK complaints. Exploring practitioner motivations and clinical reasoning, along with patient experiences associated with treating non-MSK complaints will assist in understanding why patients seek care for non-MSK complaints and how osteopathy may be best placed to contribute to multi- or inter-professional care for them.

## Data Availability

The datasets analysed during the current study are not publicly available as the authors do not have the authority to disseminate the data. The dataset is available through reasonable request at [arccim@uts.edu.au](mailto: arccim@uts.edu.au).

## Abbreviations

MSK: Musculoskeletal
non-MSK: non-musculoskeletal
NZ: New Zealand
OCF: Osteopathy in the cranial field
